# Biophysical parameters control signal transfer in spiking network

**DOI:** 10.3389/fncom.2023.1011814

**Published:** 2023-01-25

**Authors:** Tomás Garnier Artiñano, Vafa Andalibi, Iiris Atula, Matteo Maestri, Simo Vanni

**Affiliations:** ^1^Helsinki University Hospital (HUS) Neurocenter, Neurology, Helsinki University Hospital, Helsinki, Finland; ^2^Department of Neurosciences, Clinicum, University of Helsinki, Helsinki, Finland; ^3^Department of Computer Science, Indiana University Bloomington, Bloomington, IN, United States; ^4^Department of Biomedical and Neuromotor Sciences, University of Bologna, Bologna, Italy; ^5^Department of Physiology, Medicum, University of Helsinki, Helsinki, Finland

**Keywords:** microcircuit, spiking network model, neural coding, predictive coding, classification

## Abstract

**Introduction:**

Information transmission and representation in both natural and artificial networks is dependent on connectivity between units. Biological neurons, in addition, modulate synaptic dynamics and post-synaptic membrane properties, but how these relate to information transmission in a population of neurons is still poorly understood. A recent study investigated local learning rules and showed how a spiking neural network can learn to represent continuous signals. Our study builds on their model to explore how basic membrane properties and synaptic delays affect information transfer.

**Methods:**

The system consisted of three input and output units and a hidden layer of 300 excitatory and 75 inhibitory leaky integrate-and-fire (LIF) or adaptive integrate-and-fire (AdEx) units. After optimizing the connectivity to accurately replicate the input patterns in the output units, we transformed the model to more biologically accurate units and included synaptic delay and concurrent action potential generation in distinct neurons. We examined three different parameter regimes which comprised either identical physiological values for both excitatory and inhibitory units (Comrade), more biologically accurate values (Bacon), or the Comrade regime whose output units were optimized for low reconstruction error (HiFi). We evaluated information transmission and classification accuracy of the network with four distinct metrics: coherence, Granger causality, transfer entropy, and reconstruction error.

**Results:**

Biophysical parameters showed a major impact on information transfer metrics. The classification was surprisingly robust, surviving very low firing and information rates, whereas information transmission overall and particularly low reconstruction error were more dependent on higher firing rates in LIF units. In AdEx units, the firing rates were lower and less information was transferred, but interestingly the highest information transmission rates were no longer overlapping with the highest firing rates.

**Discussion:**

Our findings can be reflected on the predictive coding theory of the cerebral cortex and may suggest information transfer qualities as a phenomenological quality of biological cells.

## 1. Introduction

How sensory signals are processed by the cerebral cortex to generate relevant behavior remains an open question. Hypothetical mechanisms of these biological computations have been searched for in multiple theoretical studies (Grossberg, 1980; Mumford, 1992; Rao and Ballard, 1999; Baddeley, 2000; Barlow, 2001; Friston, 2010; Panzeri et al., 2015). Recapitulating these theories, sensory systems maximize discriminable states, or representations, given the available resources. In other words, the system optimizes decoding by finding causes, hypotheses, or predictions of the input. When a match between input and an expectation is reached, the system builds a resonant state and avoids surprises by minimizing free energy or, alternatively, represents information with economy of space, weight, and energy.

Although the nature of the code itself is unknown, biological evidence shows learning is key to generating sensory representations and complex behavior (Buonomano and Merzenich, 1998; Destexhe and Marder, 2004; Pascual-Leone et al., 2005). Given the ability of a neural system with a non-linear transfer function to compute any function (Hornik et al., 1989), it has become possible to teach a spiking network to follow and decode arbitrary noise patterns (Brendel et al., 2020, referred as Brendel model below). The significance of the Brendel model emerge from the computational interpretation of membrane voltage as an error signal from predicted input, thus directly linking the predictive coding model to biophysical parameters (Denève et al., 2017).

**FIGURE 1:**
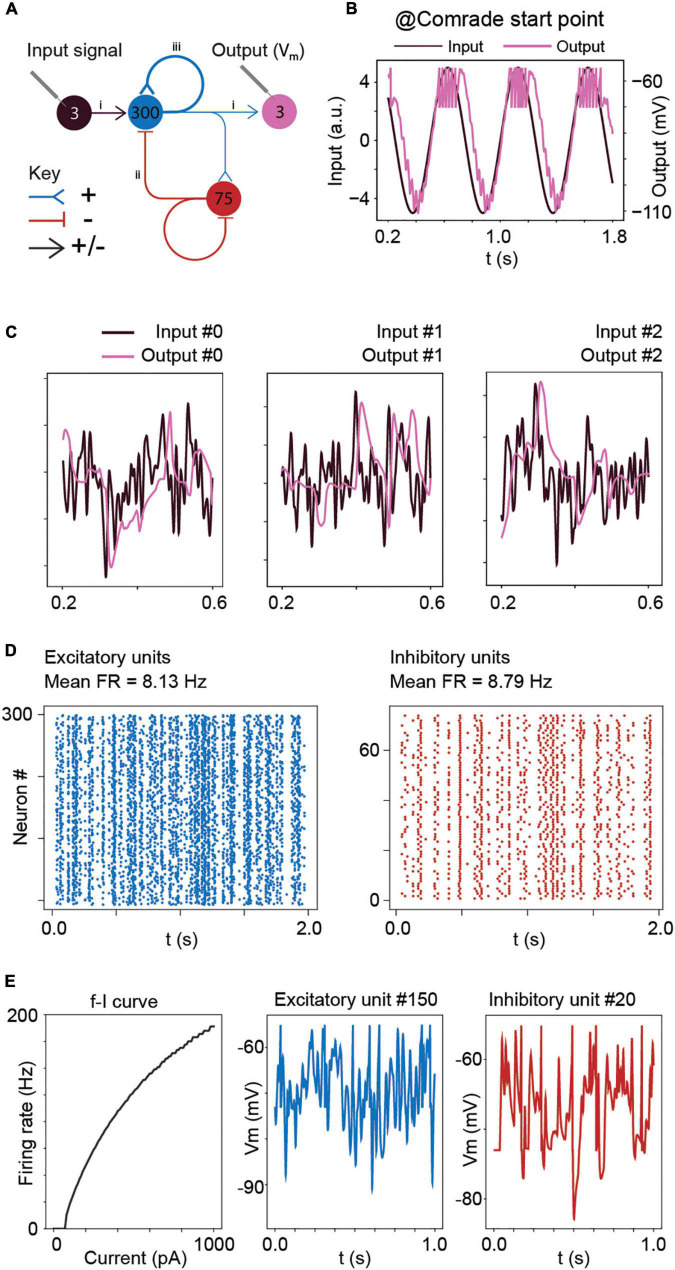
Model structure and performance at Comrade unit class after learning optimal connections with the Brendel model. **(A)** Model structure. Input consisted of Gaussian noise which was injected as de- and hyperpolarizing currents to all excitatory units. The action potential output from the excitatory units to the three output units contain both positive and negative weights. Other connections (EE, EI, II, IE) are either de- or hyperpolarizing, but not both. Blue = excitatory units and pathways; red = inhibitory units and pathways; arrowhead = signals are both positive and negative; T line end: hyperpolarizing connection; reverse arrowhead: depolarizing connection. **(B)** Sinusoidal input and corresponding output unit activity at Comrade class search start point (Table 1). The output reaches action potential threshold at the peak. The first and last 200 ms of the 2-s simulation time are omitted to avoid edge effects. **(C)** Three concurrent smoothed Gaussian noise stimuli at input units (gray) are clearly separable in the three output unit membrane voltages (black). Most high-frequency deflections are lost, resulting in clearly separate but not very accurate replication of the input. **(D)** Spiking activity in the excitatory and inhibitory units show modest firing rates for the three Gaussian noise stimuli. **(E)** f–I curve for Comrade units and representative excitatory (blue) and inhibitory (red) neurones membrane potential dynamics.

We build on the Brendel model after the training was finished and connections were fixed (Figure 1) and asked how basic membrane characteristics and the synaptic delay change information transfer and representation. Such a simple system provides an optimal window to capture extra-synaptic effects on information flow because it has minimal unintended non-linearities.

**TABLE 1:** Search ranges of the physiological parameters.

Parameter	Inhibitory	Excitatory
Capacitance (pF)	30—270 (10)	30—130 (10)
Leak conductance (nS)	1—28 (1)	0.5—15 (1)
Leak equilibrium potential (mV)	−85— −35 (5)	−85— −20 (5)
AP threshold (mV)	−65— −15 (3)	−67— −35 (3)
Synaptic delay (ms)	0.5—25 (0.25)	0.5—25 (0.25)

The values were selected to cover most of the dynamic regime. The values show range min–range max (step size). All three unit-classes were searched with the same values. During search, the same Synaptic delay value was applied to EE, EI, II, and IE connections, while the E to output unit delay was fixed (3 ms for Comrade and Bacon, and 1 ms for the HiFi unit-class).

We used four distinct signal transmission metrics housing mutually complementary features. Our first metric is *coherence* which is widely used in the signal analysis as metric for linear relationship between two analog signals (Gardner, 1992). It is typically applied with spectral analysis, allowing natural division of signal transfer into spectral components. Our second metric is *Granger causality* which, as transfer entropy, has an information-theoretical interpretation (Barnett et al., 2009; Bossomaier et al., 2016). Granger causality assumes that cause precedes its effect and that the cause contains information about the effect that is unique to the cause. While Granger causality is the computationally heaviest of our metrics and sometimes numerically unstable, it can in practice be applied to much longer delays than the transfer entropy. Our third metric, *transfer entropy*, is an information theoretical metric that measures the directed exchange of information between two systems (Schreiber, 2000; Bossomaier et al., 2016). In contrast to mutual information, which is widely used in neuroscience, transfer entropy has a direction, and it can separate the exchange of information from the effects of common history. It is strongly limited by large dimensionality and thus in this study, we were limited to a single time point history for input (optimally shifted in time) and output. Our fourth and final metric, *normalized reconstruction error*, is based on Brendel model error metric. It is a simple measure of similarity of the waveforms between input and output and is very sensitive to temporal delays.

## 2. Model

### 2.1. Base model and training

Supplementary table describes our model according to Nordlie et al. (2009). Our model (Figure 1A) followed the overall structure of Brendel et al. (2020) model. It consisted of a network of 300 excitatory and 75 inhibitory leaky integrate-and-fire (LIF) or adaptive exponential integrate-and-fire (AdEx) units which were fully connected within and between groups. In contrast to Brendel model, our three decoding units were LIF units for both the LIF and AdEx simulations. We fed the network three temporally low-pass filtered (Gaussian filter with standard deviation of 3 ms) white noise signals with Gaussian distribution of amplitude values. The three input signals were injected as currents to all the 300 excitatory units. The three output units received their input from all the excitatory units. The connection weights from input to excitatory and from excitatory to output units had both positive and negative values, necessary to capture both positive and negative deviations from the baseline. The connections within and between the inhibitory and excitatory groups were all positive.

Connection weights were learned using the Brendel model’s simulation code that is written in Matlab^®^ and is publicly available on Github^1^. As in the original code, we used time step 0.0001, membrane time constant of 50 time points, integration constant for feedforward input to excitatory population 300 time points, integration constant from excitatory to inhibitory population 50 time points, learning rate 0.00001 for learning the input to excitatory and excitatory to inhibitory connections, learning rate of 0.0001 for EE, II, and IE plasticity. The feedforward weights were generated from random normal distribution and normalized to sum of one. The initial connection weights within and between the excitatory (Exc) and inhibitory (Inh) groups were: Exc => Exc zero, except autapses −0.02; Exc => Inh every Inh neuron received connections from four Exc neurons with weights 0.5, others zero; Inh => Inh zero, except autapses −0.5; Inh => Exc every inhibitory unit connected to four excitatory units with weights −0.15, others zero. The action potential thresholds were set to half of the norm of the feedforward weights.

After learning, these weights were transferred into our more physiological model. To achieve a dynamic system with synaptic conductances at the nanosiemens scale, connection weights, except connections from the excitatory to output group, were scaled with a factor of 10^–7^.

The Brendel model calculated the decoding connections from the excitatory group to the output group by a linear readout model:


(1) $${\hat{x}}_{j}\left(t\right)=\sum_{k=1}^{N}D_{jk}r_{k}\left(t\right)$$

where ${\hat{x}}_{j}\left(t\right)$ is the estimated readout at output unit *j* at time *t, D*_*jk*_ is the decoding weight between the *k*-th excitatory unit and the *j*-th output unit, and *r*_*k*_ (*t*) is the filtered spike train on the neuron receiving the signal. We copied these connections and scaled them with 3 * 10^–8^ to have the output reach maximum dynamic membrane voltage range but with a very low number of spikes. This membrane voltage constitutes our readout trace which was then compared to input.

### 2.2. Current model and parameter exploration

Simulations were run in CxSystem2 (Andalibi et al., 2019) with a LIF model:


(2) $$\frac{dv_{m}}{dt}=\frac{-gL\left(v_{m}-EL\right)+g_{e}V_{unit}-g_{i}V_{unit}+I_{ext}\left(t,i\right)}{C}$$

where *C* denotes the membrane capacitance, *vm* the membrane voltage, *EL* is the leak equilibrium potential, *gL* is the leak conductance, *g*_*e*_ is the excitatory conductance, *g*_*i*_ is the inhibitory conductance, which is inverted by the – sign, and *Iext (t,i)* is the input signal current injection (excitatory neurons only) and where the *t* is time and *i* the unit. To avoid too large deviation from the Brendel model, neither excitatory nor inhibitory synapses had driving forces, instead, their conductances were converted to voltages by multiplication of connection weight with the unit of membrane potential, volt. After a presynaptic action potential, the *g*_*e*_ and *g*_*i*_ dynamics followed exponential function:


(3) $$\frac{dg}{dt}=\frac{-g}{\tau }$$

Where τ is the time constant of the decay. The AdEx units followed model:


(4) $$\frac{dv_{m}}{dt}=\frac{-gL\left(v_{m}-EL\right)+{△}_{T}gLe^{\frac{v_{m}-VT}{{△}_{T}}}-w+g_{e}V_{unit}-g_{i}V_{unit}+I_{ext}\left(t,i\right)}{C}$$

Where Δ_*T*_ is the slope factor or sharpness of action potential non-linearity and VT is the action potential threshold.

The adaptation current *w* has a dynamics:


(5) $$\frac{dw}{dt}=\frac{a\left(v_{m}-EL\right)-w}{\tau_{w}}$$

Where *τ_*w*_* is the time constant and *a* represents adaptation below the spiking threshold. At each action potential, the *w* is increased by *b*, which represents spike-triggered adaptation.

#### 2.2.1. Comrade units

We had three physiological regimes which we call unit-classes, denoting the initial sets of parameters in multidimensional space (Supplementary table, Unit-Classes). We first created a unit-class where both inhibitory and excitatory units had identical physiological values to mimic the units found in the Brendel model. Since the parameter values of both inhibitory and excitatory units were equal, this unit-class was called the Comrade class.

#### 2.2.2. Bacon units

We then constructed a second unit-class using physiological values from the literature. Since the unit-class was made from *empirical* experimental data this unit-class was called *Bacon* class, named after Sir Francis Bacon, the father of empiricism. The parameters were based on experimental data collected from the macaque cortex (Povysheva et al., 2013; Luebke et al., 2015; Gilman et al., 2017). The Bacon excitatory unit values for capacitance and leak conductance were derived from membrane time constant and membrane resistance. These, and action potential threshold, were extracted as mean values between Gilman et al. (2017) and Luebke et al. (2015) who studied area V1 pyramidal cells in macaque monkeys. The corresponding inhibitory unit values were the basket cell values from Povysheva et al. who studied macaque prefrontal cortex. The equilibrium potential value was based on Amatrudo et al. (2012) who fitted model parameters to structural and electrophysiological macaque V1 neuron data. Some missing LIF parameters followed earlier simulation studies (Diesmann et al., 1999; Hokkanen et al., 2019).

#### 2.2.3. HiFi units

HiFi and Comrade units were identical, excluding the output units. The HiFi output units had a much shorter synaptic delay (3 ms => 1 ms) and a higher leak conductance (4 nS => 40 nS) than the Comrade or Bacon units. These physiological changes allowed for a higher fidelity when reconstructing the input signal.

#### 2.2.4. Parameter search

We first explored a wide range of physiological parameters around each unit class to determine the dynamic regime of the system (data not shown). Parameters were then explored in detail in this narrowed dynamic range. Table 1 shows the search ranges and step sizes of the physiological parameters. Note that the three unit-classes’ starting points were not in the middle of the search spaces. The searches were two-dimensional for capacitance, leak conductance, leak equilibrium potential, and action potential threshold; the first dimension for the inhibitory, and the second for the excitatory units. The synaptic delay within and between the inhibitory and excitatory unit groups was searched in one dimension, i.e., all four (EE, EI, II, IE) connection delays varied together.

#### 2.2.5. Computational implementation

The duration of each simulation was 2 s, at 0.1 ms resolution. The first and last 200 ms were omitted for response stability, resulting in 16,000 samples for further analysis. Each main simulation round comprised of 30,000 simulations (3 unit-classes × 5 parameters × about 200 parameter combinations × 10 iterations with independent noise). Altogether, we ran about 400,000 simulations.

Simulations were computed on a workstation equipped with an Intel Xeon Processor (E5 2640–2.6 GHz), 128 GB of DDR4 memory, and one NVIDIA GK104GL (Quadro k4200) graphic card with 4GB video memory. The workstation ran the Linux operating system on a SATA III Solid State Drive.

We used the CxSystem2 cortical simulation framework (Andalibi et al., 2019; Hokkanen et al., 2019) which has been written on top of the Python-based Brian2 simulator (Goodman and Brette, 2009) mainly with the purpose of flexible model construction at a higher abstraction level and parallel parameter search.

**FIGURE 2:**
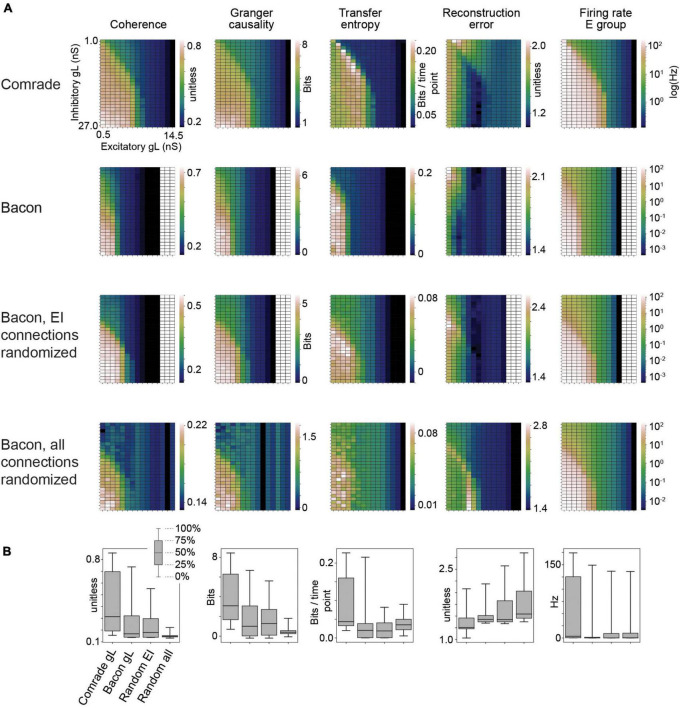
Information transfer between input and output for a range of leak conductance (gL) values in both inhibitory and excitatory units. **(A)** The first row shows coherence, Granger causality, transfer entropy and reconstruction error values as a function of varying gL at the Comrade units. Overall, information transfer is better at higher firing rates (rightmost column), although both transfer entropy and reconstruction error show a performance dip at the highest rates. The second row shows corresponding data for the Bacon units. Performance is similar to Comrade units. The third row shows data for Bacon units, when E-E, E-I, I-I, and I-E connections are randomly permuted, essentially losing the learned optimization but preserving the overall connection strength. Firing rate dependence of information transfer is preserved, but the overall level of information transfer is reduced. The fourth row shows data for Bacon units when all EI connections, current injection from input to E units as well as output from E units to output units are randomly permuted. Some information is still transferred, but the overall level is further reduced. **(B)** A boxplot across the data in panel **(A)**. The insert applies to all boxplots in this study: medians (black lines), 25 and 75% quartiles (gray rectangles) and full range (whiskers) of the data.

**FIGURE 3:**
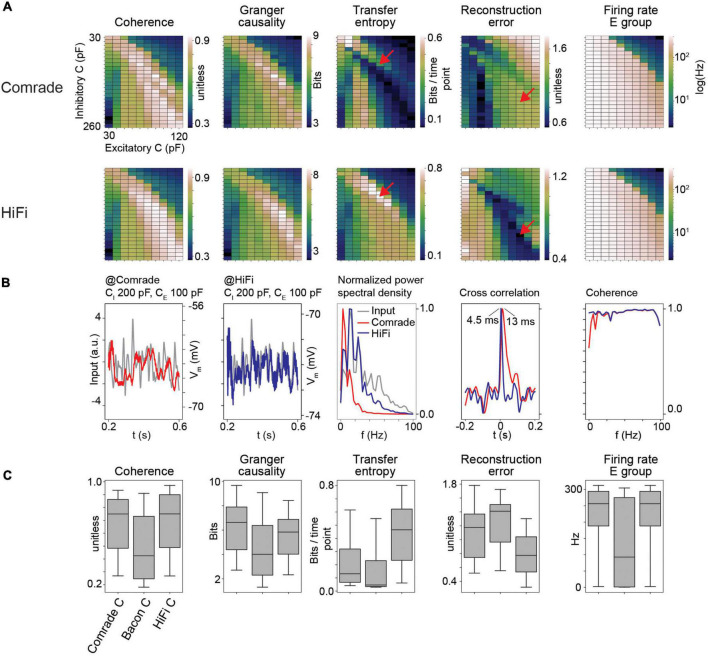
HiFi units can reconstruct the input accurately. **(A)** Information transfer as a function of varying capacitance (C) at Comrade and HiFi units. The red arrows show the maximum TE and minimum RE for HiFi units, and the corresponding positions for the Comrade. **(B)** Comrade (red) and HiFi (blue) simulation output at the capacitance values which minimize the reconstruction error for HiFi (C_I_ = 200 pF, C_E_ = 100 pF). **(C)** Each boxplot contains 240 datapoints (2D search displayed in panel **A**), and each datapoint is the average of 10 iterations with distinct input noise pattern.

**FIGURE 4:**
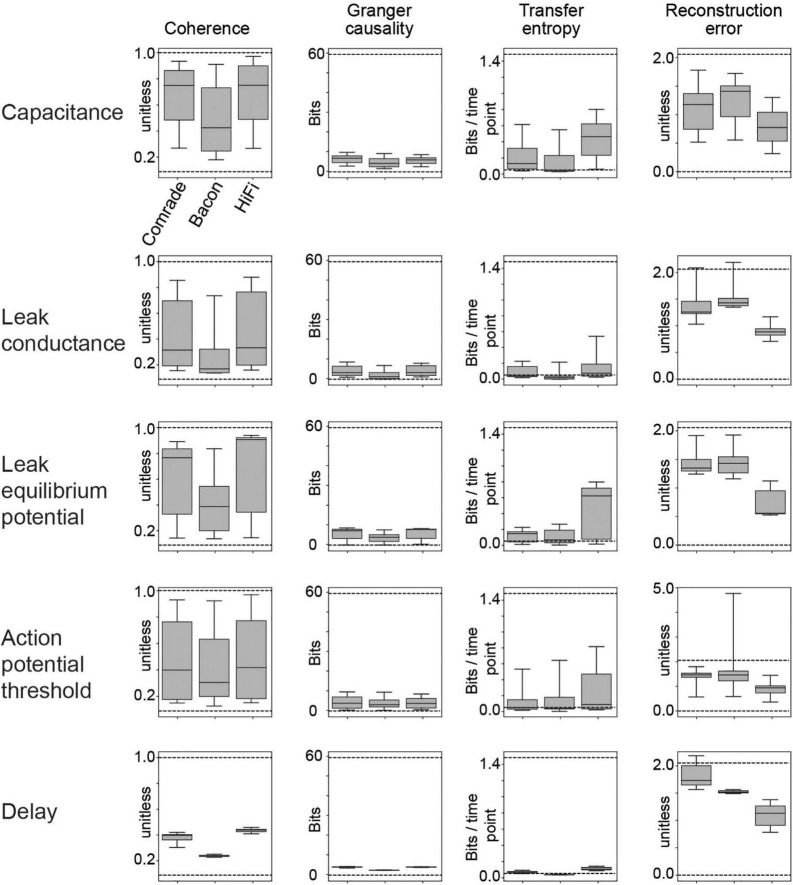
Dynamic range of information transfer attainable with physiological parameter variation. The dashed lines depict the best information transfer rates (referred as *optimal* in the text; input compared to itself as output at varying delays) or the worst rates (*unfit*; input compared to other inputs as outputs).

**FIGURE 5:**
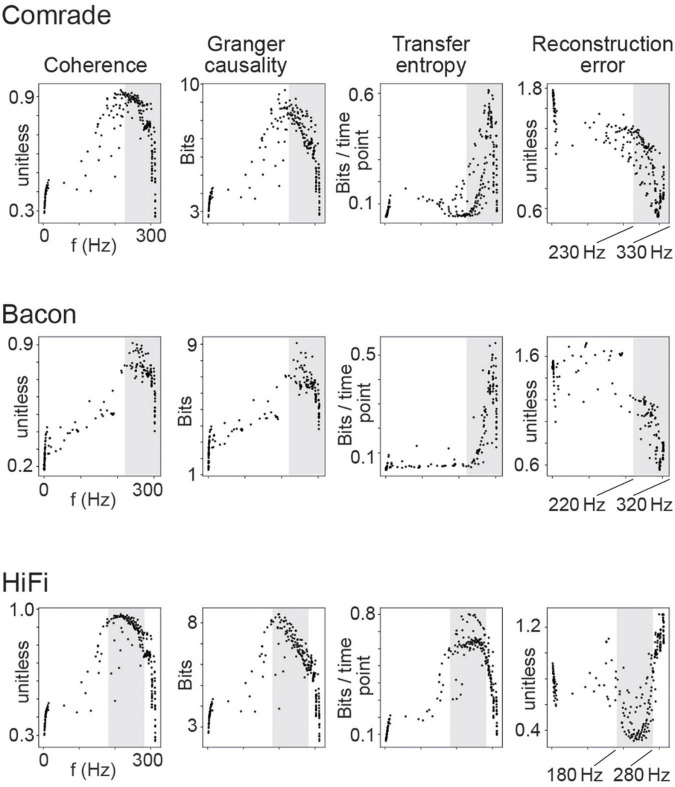
Information transfer as a function of firing rate for the 2D search on capacitance. Each dot is the average of 10 simulations with distinct inputs. The dots depict distinct combinations of inhibitory and excitatory unit capacitance. The gray shadings indicate the firing rate ranges where *TE* and *RE* reach their best values, separately for each unit-class.

**FIGURE 6:**
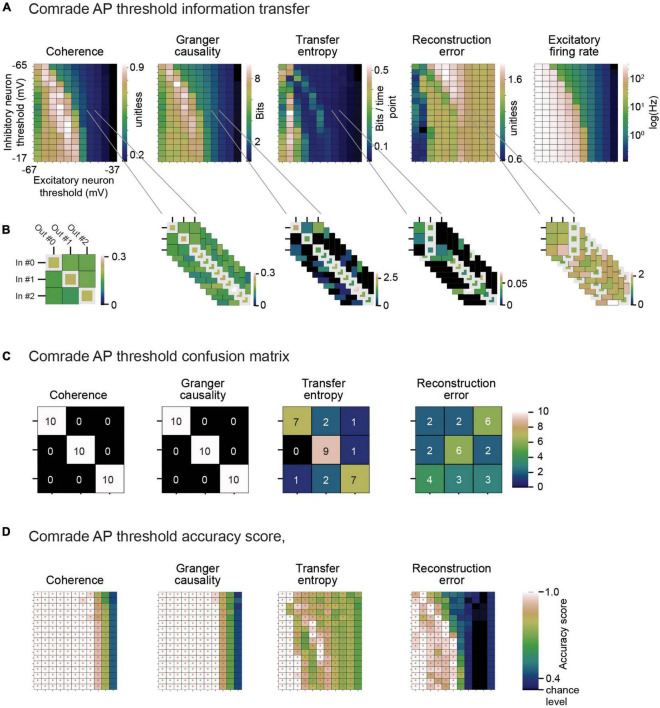
Classification across the 2D search on VT. **(A)** Information metrics. **(B)** Classification between inputs and outputs from the information metric. Example selection of ap threshold values (inhibitory unit –44 mV, excitatory unit –46 mV) lead to moderate mean firing rates (inhibitory unit 0.15 Hz, excitatory unit 0.93 Hz). On the left. The learned connectivity should match the three input patterns to corresponding output unit membrane potential patterns. For each input (row) the highest coherence value (white rectangle) indeed matches the correct input to correct output. *On the right*. Ten iterations of simulated data for different noise patterns provide 30 choices for each information metric (max for coherence, Granger causality and transfer entropy, min for reconstruction error). **(C)** Confusion matrix for calculating accuracy score. Coherence and Granger causality maxima land always at the matching input-output pairs, while transfer entropy have some failures. Reconstruction errors have more failures than hits. **(D)** Accuracy score for each information metric and each ap threshold value pair. The red asterisks depict *p* < 0.05, Bonferroni corrected for the *N* parameter combinations in the current search (*N* inhibitory ap threshold values × *N* excitatory ap threshold values).

**FIGURE 7:**
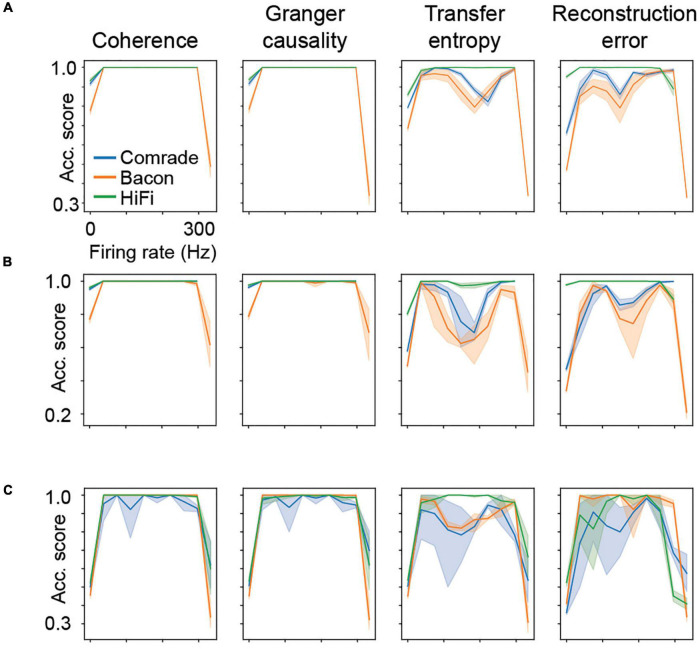
Classification accuracy as a function of firing rate. Results from all five parameter searches are averaged. **(A)** The main experiment with three inputs and three outputs, mean of 10 iterations (thick line) and the 95% confidence interval by bootstrapping (shading). Data is binned to 10 distinct firing rates. **(B)** A control experiment with six inputs and outputs, 10 iterations as in the main experiment. **(C)** A control experiment with one-dimensional searches across C, gL, EL and VT, (gL and EL augmented by two values of capacitance) in such a way that each search reaches zero and fully saturated firing rates.

**FIGURE 8:**
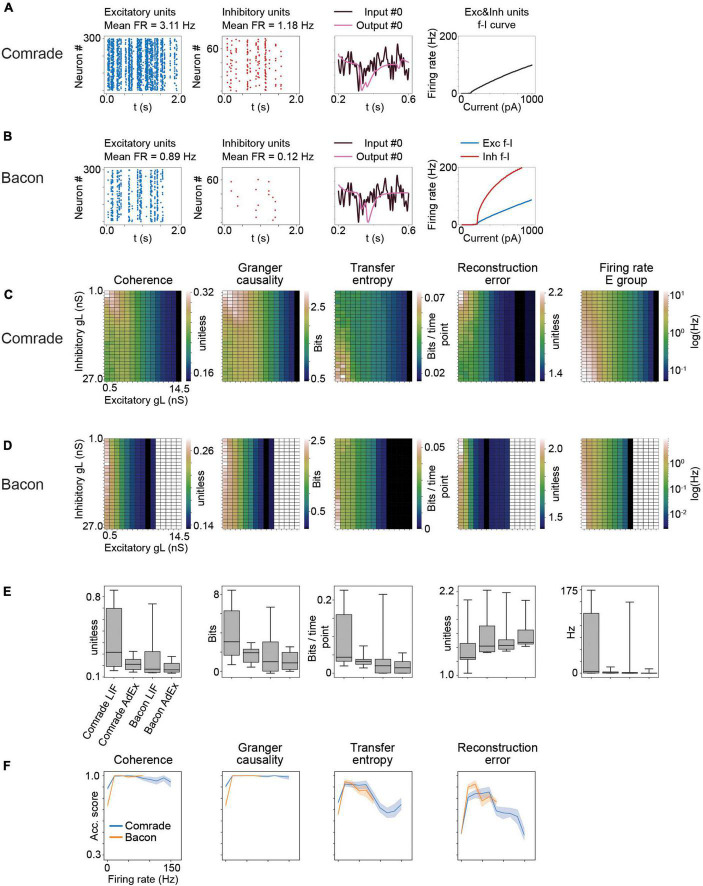
Results for AdEx model units. **(A)** The adaptation in Comrade units lead to low FR (see Figure 1D), a low-fidelity representation of the input (3^rd^ column) and to a shallow f_I slope. **(B)** As panel **(A)** but for Bacon units. **(C)** 2-dimensional parameter search for gL and resulting coherence, Granger causality, transfer entropy and reconstruction error values for the Comrade units. **(D)** Same as panel **(C)**, but for Bacon units. The system cease firing when excitatory unit gL reach 10.5 nS. **(E)** A box-plot comparing information transfer quantities and firing rates for LIF and AdEx models for Comrade and Bacon units. **(F)** Classification accuracy as a function of firing rate for Comrade (blue) and Bacon (orange) units.

The analyses, visualizations and automation for iterative runs were performed using in-house developed SystemTools software^2^. This software is publicly available and includes detailed installation instructions and jupyter notebook files for this study. The jupyter notebooks are available for Figures 1B–D, 2–6A,D, 7A, 8. The heavier simulations (LIF, AdEx, controls with randomized connectivity) were pre-calculated for the notebooks. The code to recalculating these are included into the SystemTools software, and authors are happy to provide further assistance for interested readers.

## 3. Evaluation of information transfer

### 3.1. Signal transfer metrics

The results show the mean of the matching input-output pairs (mean of #0 input to #0 output, #1 to #1, and #2 to #2) for all the four metrics.

#### 3.1.1. Coherence

For time-series analysis, coherence is used to describe a linear association strength between two data sets whose dependency can be shifted in time. We calculated coherence values between all input and output pairs as:


(6) $$C_{xy}=\frac{{\left|P_{xy}\right|}^{2}}{P_{xx}P_{yy}}$$

where P_*xx*_ and P_*yy*_ are power spectral density estimates for X and Y (the input and output, respectively) and P_*xy*_ is the cross-spectral density estimate of X and Y.

Best latency was the argmax of crosscorrelation between input and output. This was limited on positive latencies, i.e., input preceding output. Both coherence and crosscorrelation were calculated with SciPy signal package (Virtanen et al., 2020). We report the mean of coherence values from 0 to 100 Hz, sampled at 3-Hz intervals.

#### 3.1.2. Granger causality

Granger causality can be used to test if the values in time series X forecast the values of time series Y–that is to say if X Granger-causes Y (Granger, 1969; Geweke, 1982; Barnett et al., 2009). Specifically, we test if adding optimally selected and weighed values from input signal X improves our prediction of output signal Y compared to a model in which we only use past values of Y. We pick optimal linear coefficients α_*i*_ for eachY_*t*−1_,Y_*t*−2_, …, *Y*_*t*−*n*_1__ and similarly, we select optimal linear coefficients β_*i*_ for each X_*t*−p_, X_*t*−(p + 1)_, X_*t*_−(p + 2), …, *X*_*t*−*n*_2__. The null hypothesis states that adding X to our model does not improve our prediction:


$$H_{0}:\beta_{1}=0,\beta_{2}=0,\ldots ,\beta_{n}=0$$

To calculate Granger causality, our data was first downsampled by a factor of 40. Such downsampling brings successive samples closer to significant information transmission latencies, necessary for a successful evaluation of Granger causality (Lionel Barnett, personal communication). Next, each value was subtracted from its previous value to make the time series stationary. The lag order was picked by Akaike information criterion (McQuarrie and Tsai, 1998). Max lag was restricted to 100 ms for the main experiment, and the majority of realized lags were between 20 and 90 ms.

We then fit the data with a vector autoregressive model. We compared a univariate model:


(7) $$Y_{t}=\sum_{i=1}^{n_{1}}\alpha_{i}Y_{t-i}+v_{t}$$

to a bivariate model:


(8) $$Y_{t}=\sum_{i=1}^{n_{1}}\alpha_{i}Y_{t-i}+\sum_{i=p}^{n_{2}}\beta_{i}X_{t-i}+u_{t}$$

by calculating the variances of the residual terms *v* and *u* and plugging them into the *F*-statistic:


(9) $$\text{F}=\log\left\{\frac{var\left(v\right)}{var\left(u\right)}\right\}$$

and then calculating the *p*-value. We further interpreted the vector autoregressive model as information in bits by taking the base 2 logarithm of the *F* value.

Granger causality is directly affected by the variance of the residual terms, whereas information-theoretic measures only consider the probability of such deviations. This means that Granger causality is more sensitive and better suited to situations where considering the absolute values of data is important. However, if the variables are produced in a non-linear process, relying on linear Granger causality might not be justified.

While in principle Granger causality does not measure information but the difference in strength of prediction between two models, it does have an information-theoretic interpretation if the residuals are normally distributed: in that case the *F*-statistic is equal to continuous transfer entropy up to a factor of two (Barnett et al., 2009).

#### 3.1.3. Transfer entropy

Transfer entropy is designed to measure the directed, asymmetric information flow from one time-dependent variable to another. It can be understood as the conditional mutual information between past values of time series *X* and the predicted value *Y*_*t*+1_ in time series *Y*, when we already know the past values of *Y*. Formally, it is defined as follows (Bossomaier et al., 2016):


(10) $$\begin{array}{c} \begin{array}{l} T_{X\to Y}=MI\left(Y_{t+1};X_{t}^{\left(k\right)}|Y_{t}^{\left(l\right)}\right)\\ =\sum_{y_{t+1},y_{t}^{\left(k\right)},x_{t}^{\left(l\right)}}p(y_{t+1},y_{t}^{\left(k\right)},x_{t}^{\left(l\right)})log\frac{p\left(y_{t+1}|y_{t}^{\left(k\right)},x_{t}^{\left(l\right)}\right)}{p\left(y_{t+1}|y_{t}^{\left(k\right)}\right)} \end{array} \end{array}$$

To calculate an estimate for transfer entropy, the input signal was first shifted in time to the point where optimal cross-correlation was found between the input and the output. After the time shift *x*_*p*_ corresponds to *y*_*t*_. As with Granger causality, data was then downsampled by the factor of 40, leaving us with 400 observations in both the input and output signals. Each value was subtracted from its previous value to make the time series stationary. Embedding dimensions *k* and *l* were set to *k* = 1 and *l* = 1 to limit the number of possible combinations in the data. The continuous amplitude values were quantized into four fixed-length bins and each signal value was rounded to the nearest discrete value. Choice of bin number *n* follows the formula $n=\sqrt[{k+l+1}]{N/5}=\sqrt[{3}]{400/5}\approx 4$, where *N* is the sample size.

We formed a three-dimensional 4 × 4 × 4 matrix where each cell corresponded to a discrete three-dimensional vector (*Y*_*t*+1_, Y_*t*_, *X_*p*_*). We iterated through the time series and increased the observation counter by one in the cell that corresponded to the observed vector of each step.

Next, we estimated two conditional probabilities from this matrix:


(11) $$\begin{array}{l} P\left(Y_{t+1}=y_{t+1}\text{|}Y_{t}=y_{t}\right)\;and\\ P\left(Y_{t+1}=y_{t+1}\text{|}Y_{t}=y_{t}\cap X_{p}=x_{p}\right) \end{array}$$

where *y*_*t*+1_*y*_*t*_*x*_*p*_ ∈ {1, 2, 3, 4}. Estimates for entropies *H*(*Y*_*t*+1_|*Y*_*t*_) and *H*(*Y*_*t*+1_|*Y*_*t*_,*X*_*p*_) are calculated based on the estimated probabilities. Ultimately, we get:


(12) $$TE=H\left(Y_{t+1}|Y_{t}\right)-H\left(Y_{t+1}|Y_{t},X_{p}\right)$$

With four value bins, the theoretical maximum for transfer entropy is 2 bits.

The result given by this method can be interpreted as follows: a receiver observes the state of output signal *Y* at time point *t* and now has a general probability distribution for the state of *Y* at time point *t* + 1 and its entropy *H (Y).* If the receiver now observes the state of signal *X* at an optimal time point *p*, how many bits of entropy are reduced from *Y*_*t*+1_?

Unlike Granger causality, transfer entropy is not influenced by the absolute values of residual terms. It simply measures the probabilities of different observations occurring based on what we already know about previous input and output values, and the information contained in these observations based on their probabilities. Another advantage of transfer entropy is that it does not require linearity or any other assumptions about the process in which the signals were produced apart from stationarity.

However, transfer entropy requires multiple instances from each of the n^l^_*_ n^k^_*_ *n* possible value combinations to provide accurate results. In practice, this forces us to use short embedding vectors. Using longer embedding vectors would give us better results, as the state of our signal is generally determined by more than one previous lagged value.

#### 3.1.4. Normalized reconstruction error

We used a similar implementation to Brendel model, who first computed target output by leaky integration of the input; tau of the leak corresponded to the output neuron group tau, 31.25 ms for the Comrade and Bacon units and 3.125 ms for the HiFi whose output group leaked ten times more than the other classes. In contrast to Brendel model, our output has a unit (mV). To get both signals to the same space, we normalized both the target output and the simulated output to a standard scale (−1 1). Finally, the scaled target output *x*(*t*), was compared to the scaled simulated output, $\hat{x}\left(t\right)$.


(13) $$RE=\frac{\sum Var\left(x\left(t\right)-\hat{x}\left(t\right)\right)}{\sum Var\left(x\left(t\right)\right)}$$

where RE is the reconstruction error, normalized with target output variance. This gives special meaning to error value = 1; this is achieved if there are no spikes in the excitatory group, and the membrane potentials of the output group’s units stay at the resting level. Values above one may result from time shift or other inaccuracies in the system and indicate very poor replication of the input.

### 3.2. Classification performance

#### 3.2.1. From information metric to accuracy score

The original Brendel model learned to replicate the three input signals at the corresponding three output units. In the present study, we added the transformation of the model to biophysical values, the LIF model at the output units, parallel action potential generation, and synaptic delays, all without retraining, all transformations degrading the performance of the system from the optimal replication. Our information transmission metrics provide parametric values allowing the selection of the best output unit for each input. This selection results in a 3-by-3 confusion matrix. The confusion matrix allows us to use information transfer metrics to measure how accurate the network can classify each input to their corresponding output. The maximum value was used for coherence, Granger causality and transfer entropy. The minimum value was used for the reconstruction error. A separate simulation experiment used six inputs and outputs, resulting in a 6-by-6 matrix, to control for the ceiling effect in classification. In all other regards, this experiment was identical to the 3-by-3 experiment.

#### 3.2.2. Statistical testing of the accuracy

We are testing the accuracy of a classifier that tries to match input signals with the correct output signal using different criteria: coherence, Granger causality, transfer entropy, and reconstruction error. We generate random input signals *x_1_, x_2_, x_3_, … x_*i*_*, inject them into the neural simulation network and then read the outcoming signals, *y_1_, y_2_, y_3,__…_ y_*j*_.* The process is repeated *m* times, resulting in m*i trials, denoted by *n*. The synaptic connectivity in the simulation network has been converged to optimal values during training so that the normalized mean variance of the reconstruction error (*RE*) would ideally be minimized between certain input and output pairs, such that for any specific *x*_*q*_ we would get min_{_*y* ∈ *y*_1,_ *y*_2_, *y*_*j*_}*RE*(*x*_*q*_,*y*) = *y*_*l*_, *y_l_* being the “ideal pair” for that input. In this case, we have three random input signals and three output signals each round and the process is repeated ten times with different inputs, giving us *i = 3, j = 3, m = 10*, and *n = 30*.

We create a matrix *A* with *i = 3* rows representing inputs and *j = 3* columns representing outputs. The neural system places the *n = 30* observations into the matrix where the row *q* for each input *x*_*q*_ is determined by *q* ∈ {1, 2, 3} and column *o* by *f*(*x*_*q*_,*y*) = *y*_*o*_, where *f*(*x*_*q*_,*y*) seeks for the value among *y* ∈ {*y*_1,_ *y*_2_, *y*_3_} that minimizes or maximizes our criterion. The correct pair would be *y*_*o*_ = *y*_*l*_ and the correct cell *A*(*q*, *o*) = *A*(*q*, *l*). How the observations end up being distributed in the matrix depends on the ability of the system to separate the signals as well as the inputs that we generated for testing.

The accuracy score is calculated from this matrix as the ratio between the number of correctly classified signals *k* and all signals *n*. Because the data consists of repeated Bernoulli trials, all being modeled as having the same probability of successful classification *p*, the observed accuracy score follows a binomial distribution B (*n, p*).

We hypothesize that the classifier does not work and that in each trial every input has an equal probability of being matched with any of the outputs. Therefore:


(14) $$H_{0}:p=\frac{1}{j}andH_{1}:p>\frac{1}{j}$$

Under the null hypothesis, instances where the classifier’s accuracy score is higher than the expected *E*(*k*/*n*) = *p* = 1/*j* are assumed to be generated by a lucky choice of test data. In this case *KB*(30, 1/3) and we would expect to see 10 signals out of 30 being sorted correctly.

We want to know the probability of observing an accuracy score of *at least k/n* by chance alone. This probability can be directly calculated from the sum of the binomial distribution’s right tail:


(15) P(K≥k)=∑i=kn(ni)pipn-i

using, of course, *p* = 1/*j*. We choose a significance level of ∝ and reject the null hypothesis if *P*(*K* ≥ *k*) < ∝. We chose the ∝ = 0.05, Bonferroni corrected for the *N* trials in each parameter search.

## 4. Results

### 4.1. Comrade and Bacon unit classes are sensitive to membrane parameters

We studied the signal transmission properties of a trained neural network (Figure 1A) and how these properties are affected by the biophysical parameters of the network. We proceed stepwise from the Brendel et al. system, which becomes the Comrade unit class. Then we step toward biological realism with Bacon class and toward better reconstruction with HiFi class. Finally, we test the Comrade and Bacon unit classes with AdEx units.

After learning, the connections were first scaled to the nanosiemens scale to allow for adequate firing, exemplified by the network output being able to follow the sine function and only fire at the peaks (Figure 1B). Figure 1C shows the three input signals, together with the corresponding output membrane voltage. The output signals (purple) showed a poor reconstruction of the input signals (dark), with most of the high frequencies being lost, although the output signal was able to follow the overall silhouette of the input signal. The average firing frequency was within the physiological range (Sclar et al., 1990; Bakken et al., 2021) with inhibitory units firing at a slightly higher frequency than excitatory units (Figure 1D). Figure 1E shows the firing frequency of the model unit to increasing step current injections (input-frequency, or f-I curve) and representative excitatory and inhibitory unit membrane voltage traces. These results show the model, although presenting a poor reconstruction, behaves in a plausibly physiological manner. To understand the general pass-band characteristics of the network, we tested each unit class with 1–99 Hz sinusoidal inputs (data not shown). Above 30-Hz, the signal started to fail, and at 40-Hz spiking stopped for all unit classes, showing an inability to pass high-frequency regular oscillations and suggesting poor transmission of high frequencies overall.

Before examining the information transfer characteristics of our network, we sought to establish the dynamic range of the different parameters in which the network was functional (data not shown). Next, we did a parameter search (Table 1) for capacitance (C), leak conductance (gL), voltage threshold (VT) of action potential generation, leak equilibrium potential (EL), and synaptic delay (EE, EI, II, IE connections, EI below). We then observed how different information transfer metrics [coherence (*Coh*), Granger causality (*GC*), transfer entropy (*TE*), reconstruction error (*RE*)] between the input and output units, as well as the firing rate (*FR*) of excitatory units, behave as a function of the parameters. The inhibitory unit *FR* followed closely the excitatory units and thus the data was omitted below. We noted that the parameter values that maximize information transfer for each metric were somewhat different.

Figure 2 shows the 2-dimensional (inhibitory, excitatory) search results on gL values across the dynamic ranges and compares the information transmission metrics to *FR* on the right. We can see that the gL values that maximize *Coh* and *GC* were somewhat different from those of *TE*, which in turn were different from those that minimized *RE*. Overall the highest information transmission appeared at high firing rates, dipping close to saturation for all metrics, but most clearly for *TE* and *RE*. These data show that a varying leak conductance within the dynamic range of the system has a strong effect (factor of ∼5) on information transfer.

To test the relative value of connectivity versus the changes in gL, we randomly permuted the four sets of EI connections. The permutation was done within each set by randomly shuffling the post-synaptic target index, thus preserving the connection strengths in the system. The permutation only had a subtle effect on the 2D topology, improving slightly the sensitivity to gL at inhibitory units. We then permuted all connections, including EI shuffling above, but also the input to excitatory and excitatory to output units. This change led to *Coh* and *GC* metrics to collapse, significantly reducing the amount of information transfer. Figure 2B shows the boxplots summarizing the information transmission magnitudes across the 2D search. The medians were similar for the Bacon data regardless of learning, whereas the Comrade start point showed always the highest information transmission and the highest firing rate (Friedman test *p* < 0.001, *N* = 405).

These results show that randomizing local connectivity (EI) had a surprisingly subtle effect on the overall metrics, while randomizing all connections trivially collapsed the *Coh* and *GC* metrics and increased RE (random input to output relation). The paradoxical increase of TE for randomizing all connections suggests limited value of the metrics, perhaps related to non-zero spiking at high excitatory gL, the single sampling point in time or non-optimal temporal shift of the point. The varying leak conductance caused a major variation in information transfer, surpassing the variation caused by permuting the EI connectivity.

### 4.2. HiFi output units are necessary for good reconstruction

Although previous data illustrated well the important role gL values have in determining information transfer, the output signal still shows a poor reconstruction. To try to improve signal reconstruction, we optimized output units by increasing their gL from 4 to 40 nS and lowering the delay between the excitatory and output units from 3 to 1 ms without altering the middle layer. This was done to shorten the memory trace of earlier events and thus allow for fast replication of information available in the middle layer. Figure 3A shows that enabling fast response in output units results in drastically different parameter topology for *TE* and *RE* (Figure 3A, red arrows at *TE* maximum and *RE* minimum for HiFi) as well as reduction of *RE* values, while the parameter topology and range for *GC* and *Coh* are preserved. Excitatory group *FR* are identical because there is no change in the excitatory (E) group between Comrade and HiFi. This result shows that the different metrics have clearly individual characteristics with *Coh* and *GC* being most consistent.

At the minimal error for HiFi units (C_*I*_ = 200 pF, C_*E*_ = 100 pF), the Comrade unit fails to replicate the high frequencies (Figure 3B). This is clearly visible in the power spectral density, where low frequencies dominate for the Comrade output units, whereas the HiFi units follow the input at higher frequencies. There is only a minor extra lag in cross correlation peak between the input and output for Comrade, and the coherence between input and output is worse for low frequencies, compared to HiFi. These results suggest that for accurate replication of fast components in sensory data, readout neurons play a key role in the accurate reconstruction of the signal. Moreover, information transfer topology is more similar between the four metrics in HiFi than in the Comrade units, suggesting similar dependence on capacitance with faster and short-memory readout.

Figure 3C shows the magnitudes of readout metrics across the three unit-classes. The change from Comrade to HiFi improves especially *TE* and *RE*, in line with their sensitivity to fast transients; *TE* includes only one optimally shifted time point as input and output history and *RE* is very sensitive to delay (Supplementary Figure 1). For all metrics, the group comparisons were significant (Friedman test, *p* < 0.001).

Mathematically, the capacitance (Figure 3) and leak conductance (Figure 2) are not independent parameters, because they are linked by membrane time constant. They, however, provide different views to approximate membrane surface area and ionic conductances, respectively.

In a network with fixed synaptic delays between excitatory and inhibitory units, Rullán Buxó and Pillow (2020) described a back-and-forth synchronous signal propagation. Our network operates in asynchronous state (Figure 3B). This is likely influenced by the white noise input to excitatory units. However, we can not exclude temporally or spatially limited synchrony phenomena.

### 4.3. Physiological parameters cause extensive variation in information transfer

To better quantify the range of information transfer due to changes in physiological parameters, we compared the 2-dimensional parameter search results to information transfer at the optimal and least fit possible numerical transfer rates. The optimal condition comprised a copy of the input signal at the output at different delays, and the most unfit condition comprised one of the two other inputs as the output (Supplementary Figure 1). The optimal *Coh* and *GC* values were dependent on delay. *GC* was also sensitive to a maximum allowed delay in the VAR model (100 ms in this study). *RE*, again, was very sensitive to delay, reaching random levels shortly after 10 ms shift. *TE* was not sensitive to delay, because we look only at time history = 1, with optimal time shift.

Figure 4 shows the range of the information transfer in simulations against the optimal and unfit values (highest and lowest mean values in Supplementary Figure 1, upper and lower dashed lines, respectively, in Figure 4), for all five parameter searches. Varying C, gL, EL, and VT had drastic effects on *Coh*, ranging almost the full range of values. For *GC*, the current system fails to reach the optimal values, covering systematically less than 1/6 of the range. Changing the max allowed lag to 200 ms increased optimal values for *GC*, especially at longer latencies (Supplementary Figure 1, gray curve), but the *GC* values for simulated data were almost unchanged (For C, Supplementary Figure 2). *TE* reached from minimum values to half the maximum values. *RE* was poor for most Comrade and Bacon simulations (values above one), whereas HiFi reached value 0.32, indicating they were best able to accurately follow the input signal. In contrast to the other metrics, changing the synaptic delay in the EI connections from 0.5 to 25 ms had only a minimal effect on the information transfer.

### 4.4. Action potential frequency drives information transfer

We observed that for LIF units, higher frequencies were associated with better scores in every information transfer metric in all three unit-classes (Figure 5). The association was non-linear with a saturation point around 300 Hz where the information transmission started failing. Upon closer examination we realized this saturation occurs due to the immediate activation of neurons after their refractory period was over (data not shown), resulting in loss of entropy. This failure appeared first in *Coh* and *GC*, whereas the *TE* and *RE* turned from best to failure only at somewhat higher frequencies (shaded in Figure 5).

### 4.5. Robust classification in the dynamic regime

We compared the membrane potential of each output unit to the each of the input signals. From this 3-by-3 comparison, the winner was the best information transfer value. After running this analysis on 10 simulation rounds with different input signals, the analysis resulted in a confusion matrix where, ideally, each input unit would be correctly paired to its output unit 10 times.

To give an example of classification, Figure 6A shows the 2D search data on the action potential threshold (VT) for the Comrade unit class. The highest *Coh* and *GC* values are in the high firing rate (>100 Hz) regime but drop for the highest rates, as with search on C (Figure 5 top). The *TE* and *RE* have a different topology, with the best values at higher frequencies (Figure 3A). For the selected VT values (inhibitory unit −44 mV, excitatory unit −46 mV, Figure 6B), *Coh* and *GC* have great classification performances, with these two metrics being able to correctly pair every input to their output (Figure 6C) despite low mean firing rates (inhibitory unit 0.04 Hz, excitatory unit 0.93 Hz). *TE* also offers a relatively good classification performance, correctly classifying most of the simulations (accuracy score 0.8, *p* < 0.001, Bonferroni corrected for the *N* 2D search items, 95% ci 0.58–0.90). *RE* shows a poor classification performance being unable to accurately classify most input-output pairs (accuracy score 0.37, *p* > 0.05, 95% ci 0.20–0.56). From these data we can observe that classification is a robust measure for information transfer and it enables direct comparison of the different metrics.

Looking at the accuracy scores through the AP threshold values we were able to see that even in conditions of low information transfer, the network was able to generate significant accuracy scores (compare Figures 6A–D, where red dots indicate statistically significant accuracy). This robust effect could be seen in all unit-classes and membrane parameters (Figure 7). Figure 7A shows the classification accuracy score for the main experiment as a function of firing rate, with all physiological parameter searches averaged together. For *Coh* and *GC*, classification accuracy scores remain at one, excluding the first and last aggregate *FR* bins, which include very low and high *FRs*. For Bacon and Comrade units, the *TE* and *RE* values dip, in addition, at the middle *FR* ranges. To exclude that the excellent accuracy scores result from a ceiling effect, Figure 7B shows the same 2D search with 10 iterations as in the main experiment, but for six inputs and six outputs (chance rate 1/6). The results are similar to the main experiment: only the first and last *FR* bin showed a drop for the *Coh* and *GC*, while the Comrade and Bacon units replicate the mid-frequency dip for the *TE* and *RE*. Finally, Figure 7C shows the data for an extensive 1D search across the parameter variations, with the aim of better covering the zero and fully saturated *FRs*. As expected, all four metrics drop closer to random accuracy scores (1/3) at the first and last *FR* bins.

### 4.6. Adaptation leads to low-frequency code

In biological neurons, intracellular current injection to soma together with measurement of the membrane voltage have been used to characterize the response properties of a neuron. LIF model has a limited ability to replicate such membrane voltage traces, whereas relatively simple adaptive models fit considerably better (Rauch et al., 2003; Jolivet et al., 2004; Brette and Gerstner, 2005; Gerstner and Naud, 2009). Thus we next did the Comrade and Bacon simulations with AdEx units which extends the LIF model by one dynamic adaptation variable *w*, and three additional model parameters, *a, b*, and *tau*_*w*_. These model parameters were selected in such a way that the unit adaptation timing qualitatively mimicked the adaptation current of a fast spiking type II inhibitory unit and an excitatory unit (Mensi et al., 2012).

Adaptation and exponential action potential generation mechanism in Comrade units (at start point, Figure 8A) led to lower *FR* (1–3 vs. 8–9 Hz, Figure 1D), a low-fidelity representation of the input, and to a clearly less steep f-I curve than with LIF units (Figure 1E). Bacon unit firing (Figure 8B) were even more sparse. Nevertheless, the output looks like an abstraction of the input signal, albeit delayed. The f-I curves are more steep for inhibitory than excitatory units, in line with experimental data (Mensi et al., 2012).

A clear difference between AdEx and LIF units emerge for the information transfer for varying leak conductance (Figure 8C). For Comrade AdEx units, both *Coh* and *GC* show their highest values with small leak in both inhibitory and excitatory population (the left upper corner of the plot). However, the highest firing rates are at high leak in inhibitory and low leak in excitatory units (left lower corner). For LIF units the two topologies overlap, and the information transmission follows the firing rate (max at left lower corner, Figure 2A). The *TE* for AdEx is more in line with the LIF results, with best values with the highest FR. The *RE* is more difficult to interpret and the error values are consistently very high (>1). For Bacon AdEx units the system cease firing altogether with high leak in excitatory population, but otherwise there is a similar trend; the highest *Coh* and *GC* values are off the highest *FR* (Figure 8D).

Figure 8E shows that the LIF model results in systematically higher information transmission and firing rates [Wilcoxon signed rank test *p* < 0.001 between LIF and AdEx models, tested separately (*N* = 10) for each metrics plus the *FR* and Comrade and Bacon units].

Figure 8F shows that classification accuracy for *Coh* and *GC* drops only at the lowest *FR* bin, as with LIF units (Figure 7). The firing rates never exceed 150 Hz and thus there is no high-frequency saturation with accuracy drop, as with the LIF units. The *TE* and *RE* show somewhat more modest accuracies, but well above the 1/3 chance level.

## 5. Discussion

### 5.1. Accurate reconstruction is costly, classification is cheap

In the present study, we used a simple spiking network which had learned to replicate the inputs at the membrane voltage of the corresponding output units (Brendel et al., 2020). After learning, we implemented delay, parallel firing and varied the biophysical parameters of the system. We were interested in exploring how cell physiology could affect information transfer and experimenting with some key information transfer metrics. Our results show that accurately reconstructing an input signal is a difficult task for a neuronal system; only a few parameter combinations in HiFi class reached low *RE* values. This narrow parameter range combined with the expensive nature of high-frequency firing makes signal reconstruction an inefficient way to represent information. Compared to LIF, the AdEx model reduced firing rates and information transmission and disconnected the highest information transmission from the highest firing rates of the system. These differences may emerge from adaptation or from the exponential action potential mechanisms. AdEx model did not, however, improve replication of the input signal in the output units. Despite the poor reconstruction, *Coh* and *GC* indicated some degree of information transfer throughout most of the dynamic range. Consequently, even very modest firing rates showed significant classification, suggesting that classification is a more achievable task for biological systems. These results have interesting theoretical implications for biological systems as they place classification as a possible model for representation.

### 5.2. Measuring information transfer in neural systems

We selected *RE* as one of our performance metrics because it was used in the reference work (Brendel et al., 2020). It is closely related to root-mean square (RMS) metric, common in machine learning applications. The RMS metric, as well as other time-series forecasting metrics, include timepoint-by-timepoint subtraction of the data from the prediction (Caruana and Niculescu-Mizil, 2004; Cerqueira et al., 2017; Makridakis et al., 2018; Lara-Benitez et al., 2021), but in addition, the RMS metric is considered to be a good general-purpose metric also for binary classification problems (Caruana and Niculescu-Mizil, 2004). We show that *RE* is very sensitive to delays, reaching random performance in 10 ms with our input signal. However, in both macaques and humans, visual processing take place in longer timescales (Salmelin et al., 1994; Bullier, 2001) making *RE* a less than an ideal tool to study signal transfer in biological models. The need to compare computational models and experimental data makes finding and using alternative methods to analyze information transfer important.

Since the development of information theory (Shannon, 1948), various methods have been developed to quantify information content and information transfer (Amblard and Michel, 2012). The purpose of using transfer entropy as a measure for information transfer in our work is to obtain an information-theory-based measure that reflects the reduction of uncertainty in the output signal based on observations from the input. Transfer entropy has two major advantages. Firstly, the result it yields is easily interpretable and can be compared against clearly defined lower and upper bounds. Secondly, it is model-independent and does not require assumptions about the nature of interactions between the input and the output. As such it can be applied to a simulated neural system that does not transfer signals in a mathematically prespecified way. On the downside it does not describe or predict these interactions any further by suggesting a model–it simply tells if one variable can reduce entropy from the other. Transfer entropy can be understood as the decrease in the number of binary questions that one needs to ask to deduce the state of an output value after observing a single input value. In this study, transfer entropy shows how much the input can reduce the uncertainty of the resulting output. Our results show that transfer entropy is not only highly sensitive to changes in biophysical parameters, but also tenuous as an information transfer metric if, as in this work, data is limited to one time point with a time shift.

Granger causality is another widely used method for determining a predictive relationship between two signals. It measures the difference in explained variation between two linear time series models: one that only uses past values of the output itself as a predictor of its future and one that adds an input signal as a second predictor. The result it gives is a statistical test on whether this observed difference is purely random. Granger causality is in a sense more specific than transfer entropy: it assumes linearity, fits a model, and estimates how this model is affected by the input data values, not just their estimated statistical frequencies.

The idea behind Granger causality is nonetheless similar to the idea behind transfer entropy: it estimates the increase in what we know about the output based on observations about the input. Granger causality and transfer entropy are proven to be equivalent for Gaussian variables, but the equivalence does not show up in our results. This difference has probably two different origins. First, Granger causality only corresponds to continuous transfer entropy, which takes into account the reduction of entropy in the intermediate steps, whereas transfer entropy needs to be discretized (Schreiber, 2000). Second, we utilized a high lag for Granger causality; after lag order selection it captured up to 90 ms of temporal information. In contrast, the high dimensionality limited transfer entropy to a single time point. Given the more consistent results for *GC* than *TE* across the unit classes, our results show that *GC* captures the signal transmission by a neural system better than *TE*, perhaps due to the dominance of low frequencies in such transmission.

Coherence and Granger causality showed similar topology across the parameter search landscapes, and both were robust to the change in output units from Comrade to HiFi class. However, our model captures most of the available dynamic range of *Coh*, whereas only some 10–20% of *GC* information passes our model. Given that *Coh* measures synchronized oscillations, i.e., oscillations with a constant phase difference across time, our model may not pass unsynchronized oscillations, and consequently, most of the white noise input variance could be lost in our simple system. Naturally, the inability to follow sinusoidal input beyond 30-Hz at the unit-class starting points limits information from high frequencies and perhaps affects more *GC* than *Coh*. Further study should extend this finding to understand how much of the dynamic range difference between *GC* and *Coh* emerge from the inability to pass incoherent signals, does the low-pass envelope follow firing frequency, and can the incoherent or higher frequency signals perhaps pass a more natural system with variable unit properties and complex structural connectivity.

### 5.3. Implications for biological systems

#### 5.3.1. Energy efficiency

Energy efficiency is an ever-present evolutionary pressure in all organisms. Firing action potentials is an energy-demanding process, as it requires active ion transport to maintain a particular membrane potential; together with a rather small basal metabolic rate, action potentials and resulting release and recycling constitute a hefty energetic cost to gray matter (Attwell and Laughlin, 2001). At the same time, reliable and accurate signaling is important for information transfer, creating a need for the brain to encode information efficiently. Our results with an artificial neural network show that a temporally sparse code can transmit a gist of the input signal efficiently and it could also be a mechanism used by biological neuronal networks. This goes in line with sparse coding in the visual cortex (Tovee et al., 1993; Vinje and Gallant, 2000). Our results show that such a sparse code was not enough for the accurate reconstruction of the input but was highly successful at selecting a category from a fixed set, i.e., classification. This suggests that a biological neural network could prefer selection between categories, rather than accurate reconstruction of sensory inputs. This selection could manifest biologically as neuronal representations being conveyed as neuronal chains, describing how a series of signals would travel through the network. Instead of decoding the spiking response into membrane potential of the output units, as in our artificial model, decoding would manifest as neuronal chains, evolving in time.

#### 5.3.2. Information transfer profiles as a characteristic of neurons

Our results showed that information transfer metrics were strongly dependent on membrane parameter values. This insight combined with the fact that in both humans and mice different neuronal types have different morphological, transcriptomic, and electrophysiological properties (Hodge et al., 2019), implies that different cell types may have different information transfer profiles which depend on instant membrane parameters. Such information transfer characteristics could be viewed as a phenomenological characteristic of the cell. Consequently, different characteristics of information may be better transmitted by different neuronal types. Fast spiking neurons may have higher fidelity and be better at accurate representation, while slower spiking neurons may have a sparser code, and be more efficient at encoding information at distinct classes. Experimental evidence in favor of such distinction is sparse, however. Input layers to macaque monkey V1 show higher firing rates than output layers (Snodderly and Gur, 1995). Interestingly, these input layers showed *less* orientation selectivity than the output layers, which can be interpreted as input signals being dense representation of orientation whereas the output signals being able to provide a sparse representation. For inhibitory neuron types, the PV neurons help synchronize lower frequency oscillations than SST neurons (Jang et al., 2020), but these finding were linked primarily to the feedforward (PV) vs. feedback (SST) synchrony rather than to the fidelity of representation.

Another avenue for generating predictions would be to look at the types of information processed by different brain subsystems. Based on our results, one might expect neurons from regions that need accurate representations, such as the cerebellum, to have parameter values that favor accuracy and thus present higher frequencies. At the same time, regions that need more sparse information, such as the cortex, would present lower frequencies. These rough predictions match the observations of Purkinje cells in the cerebellum whose mean tonic firing rate is 23 Hz, (Fortier et al., 1993), associated with accurate timing of motor response, compared with pyramidal cells in the motor cortex (13 Hz), associated more with the initiation of behavior.

Accurately modeling these combinations of neuronal types is key for generating predictions that would further validate or disprove our hypothesis on viewing information transfer as a phenomenological characteristic of neurons. Thus, further work needs to be done in looking at how different architectures and combinations of unit classes impact information transfer.

### 5.4. Implications for models of neural systems

Our work extends the findings of Brendel et al. (2020) by showing that signal transmission is heavily influenced by the biophysical properties of model units. In biological neurons, membrane conductance is under complex regulation, and we show that such regulation affects not only the firing rate but also information transmission. In our arbitrary system, accurate reconstruction occurs best under higher firing frequencies. We consider it unlikely that more complex biological model unit or network could avoid loss of information with loss of firing rate. Given the critical importance of energy efficiency in biological brain, a coding scheme surviving also low firing frequencies could have provided ecological advantage.

**FIGURE 9:**
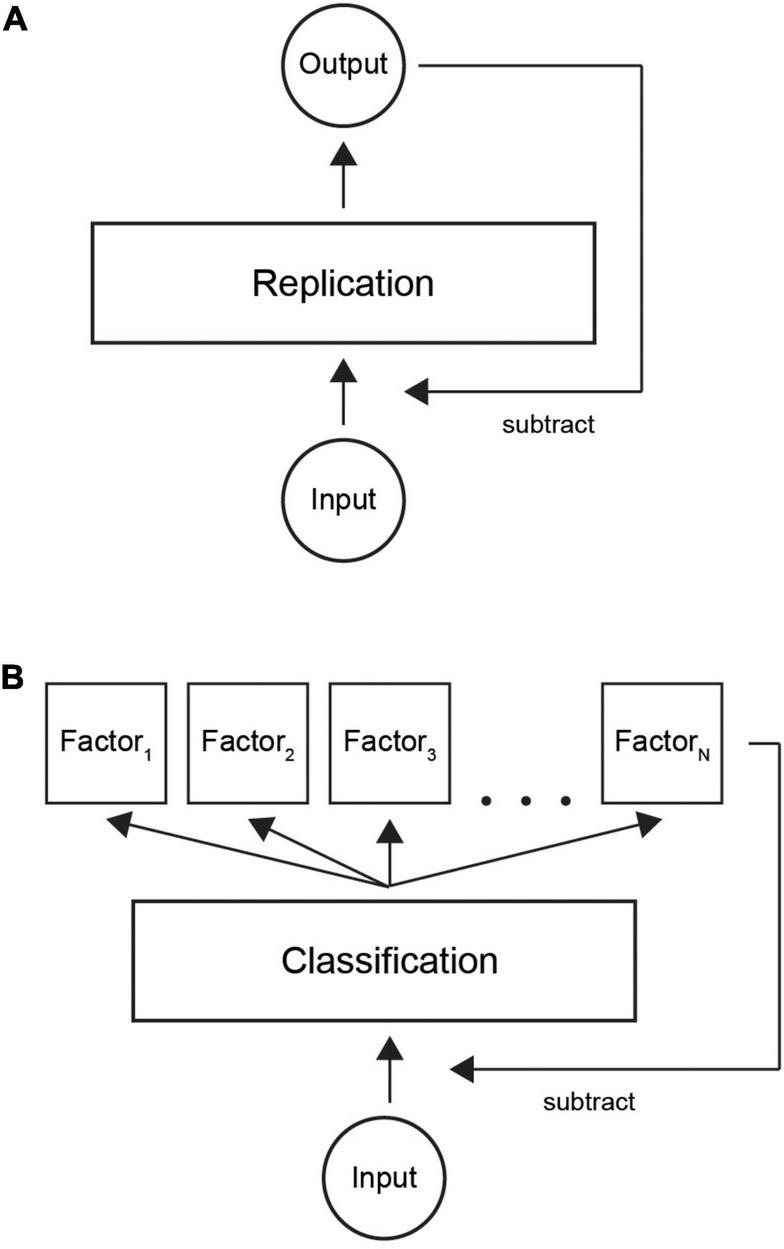
Illustration of classification with predictive coding theory. **(A)** Current model is based on replicating input by a neural network. When output is similar to incoming signals, the system represents the input and input can be attenuated. **(B)** In our refined model, the system first learns a factorial representation of incoming data, for example, in distinct neural clusters. Thereafter, classifying the input to correct neural clusters by a sparse code provides an economical way to trigger the necessary model factors. Summation over the active neural population provides the internal representation.

Predictive coding theory states that input is reconstructed by a neural system. The output is then fed back and subtracted from the input (Rao and Ballard, 1999; Friston, 2010; Clark, 2013). Figure 9A shows the basic idea of predictive coding: a layer of neurons learns to replicate the input by iteratively subtracting the network output from the input, until there is no coding error (we omit the hierarchical aspect for simplicity).

We suggest a new theoretical model where classification is used as an efficient way of encoding information (Figure 9B). First, learning factorizes input patterns to a set of internal models. Thereafter, an input gets classified into one of a finite number of factors. The resulting factorial output signal is then fed back to the input layer in a similar way to the classical model. Our model would complement the existing models by suggesting an efficient way of decoding a sparse code. In summary, we suggest an ecologically valid computational implementation of the currently prevailing predictive coding theory.

### 5.5. Future directions

The parameter values studied here are not directly applicable to biological systems given the artificial network structure and simple unit model.

In future work, it will be useful to study information transmission and representation with Hodgkin-Huxley model units and active conductances in their dendritic compartments. The Hodgkin-Huxley model would provide significantly better reconstruction of biological neural membrane, and the active dendrites are known to be central for integrating synaptic inputs and synaptic plasticity. Moreover, our network structure is far off from biological networks and, for example, feedforward inhibition is likely necessary for fast information transmission. Such work would provide an approximation of actual biological parameters, necessary for efficient information processing.

In the current work, learning was executed with the original model (Brendel et al., 2020) in Matlab whereas the simulations were implemented in the python-based CxSystem2/Brian2 framework. Future work needs to examine together the original learning model and other contemporary plasticity models (such as Clopath et al., 2010). Such reconciliation would promote teaching biologically realistic models with arbitrary data and the study of computationally functioning brain models.

## Data availability statement

Publicly available datasets were analyzed in this study. This data can be found here: Analysis, visualization and automated simulation code: https://github.com/VisualNeuroscience-UH/SystemTools. Simulation framework: https://github.com/VisualNeuroscience-UH/CxSystem2. Simulated data (516 GB): By request from the corresponding author.

## Author contributions

TGA and SV conceived the study and wrote the manuscript. TGA did the main simulations and analyses. SV programmed the SystemTools software for analysis and automated simulations. VA developed the CxSystem package in previous study, consulted on computational implementation, and proofread the manuscript. IA programmed classification analysis and conceptualized Granger causality and Transfer entropy analyses, and wrote the corresponding descriptions. MM did the simulations and analyses for the control study. All authors contributed to the article and approved the submitted version.

## Footnotes

1
https://github.com/machenslab/spikes

2
https://github.com/VisualNeuroscience-UH/SystemTools
