# Supplementary figures and images for: Biophysical parameters control signal transfer in spiking network
Source: Front Comput Neurosci. 2023 Jan 25;17:1011814. doi: 10.3389/fncom.2023.1011814 (PMC9905747; doi:10.3389/fncom.2023.1011814)

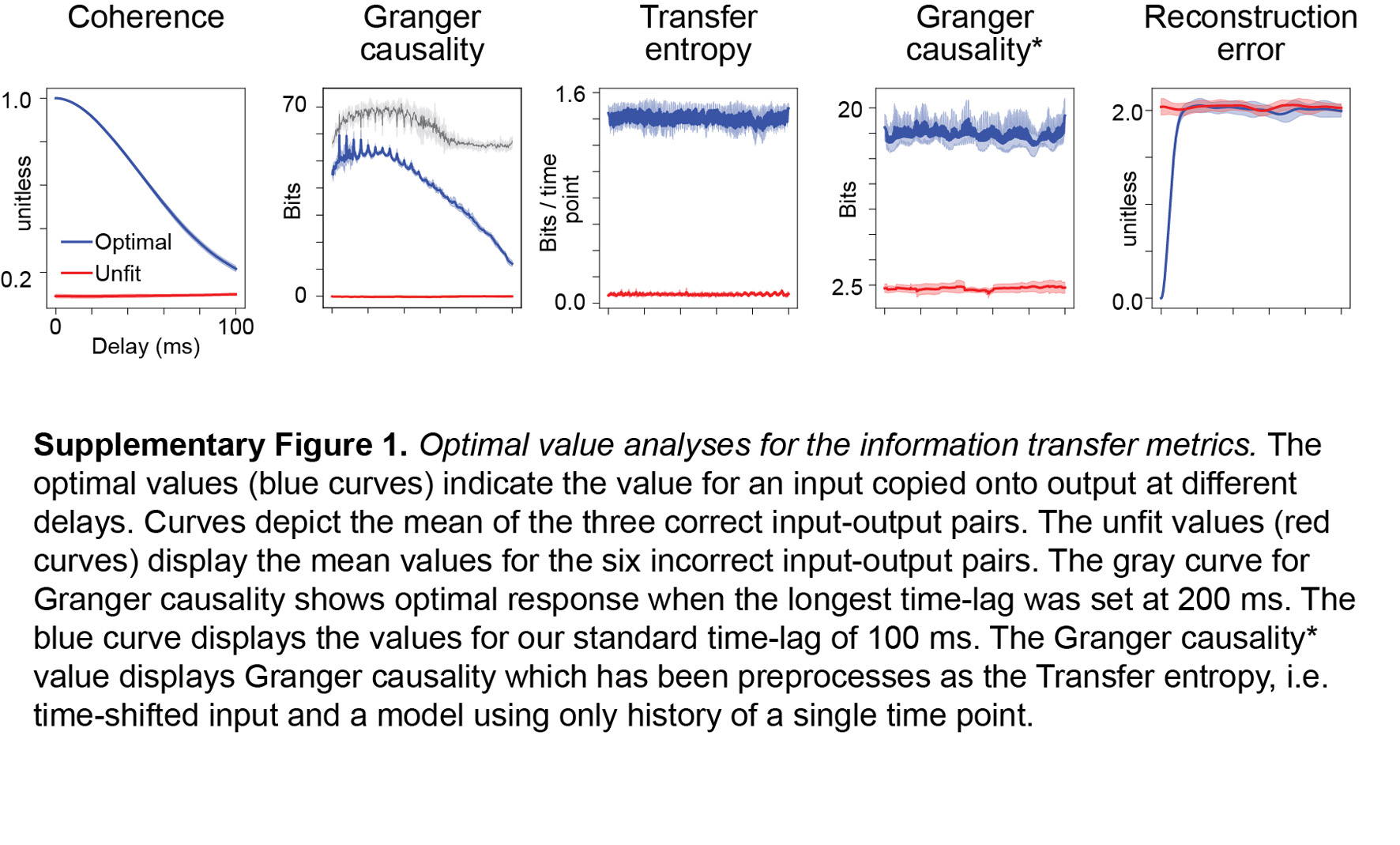

Supplement: Supplementary file 1 [file Image_1.JPEG]

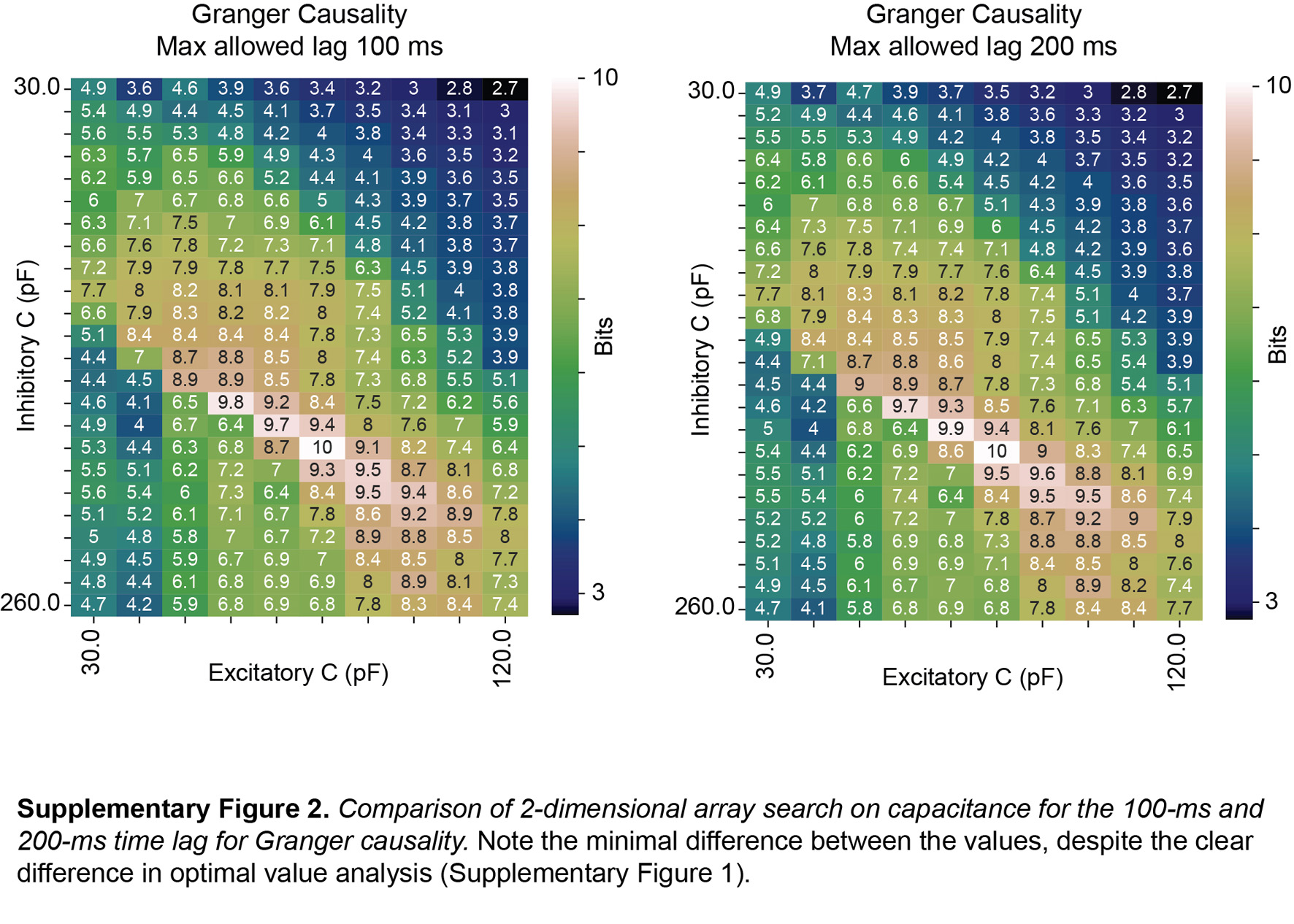

Supplement: Supplementary file 2 [file Image_2.JPEG]
