# Supplementary material for: Biophysical parameters control signal transfer in spiking network
Source: Front Comput Neurosci. 2023 Jan 25;17:1011814. doi: 10.3389/fncom.2023.1011814 (PMC9905747; doi:10.3389/fncom.2023.1011814)
Supplement: Supplementary file 3 [file Table_1.DOCX]

**Supplementary Table.** Model description according to Nordlie et al. (2009).

| Model summary | |
| --- | --- |
| Populations | Input, output, inhibitory and excitatory |
| Unit-classes | Comrade, Bacon, HiFi |
| Topology | ------ |
| Connectivity | Input to all excitatory units; all-to-all within the hidden layer; all excitatory units to output. |
| Neuron Model | Leaky Integrate-and-Fire |
| Synapse Model | Fixed connection value |
| Plasticity | Pretrained weights through the Brendel model |
| Inputs | Smoothed Gaussian noise signal |
| Measurements | coherence, transfer entropy, Granger causality, and reconstruction error |

| Unit-Classes | | | | | |
| --- | --- | --- | --- | --- | --- |
|  | Comrade | Bacon (exc.) | Bacon (inh.) | HiFi | Ref for Bacon |
| Capacitance (pF) | 125 | 36 | 86 | 125 | Povysheva et al 2013; Gilman et al 2017; Luebke et al., 2015 |
| Leak conductance (nS) | 4 | 4 | 3.9 | 4 | Povysheva et al. 2013; Gilman et al. 2017; Luebke et al., 2015 |
| Leak equilibrium Potential (mV) | -73 | -72 | -72 | -73 | Amatrudo et al 2012 |
| AP threshold (mV) | -55 | -41 | -41.8 | -55 | Povysheva et al. 2013; Gilman et al. 2017; Luebke et al., 2015 |
| Synaptic delay (ms) | 3 | 3 | 3 | 3 or 1 | ------- |

| Populations (N units) | |
| --- | --- |
| Input | 3 |
| Excitatory | 300 |
| Inhibitory | 75 |
| Output | 3 |

| Connectivity | |
| --- | --- |
| Input - E | Pre-learned, no delay, current injected to E group |
| EE, EI, IE, II | Pre-learned, delay = Synaptic delay |
| E - Output | Pre-learned, delay = 3 ms (Comrad, Bacon) or 1 ms (HiFi) |

| Neuron and Synapse model | Equation |
| --- | --- |
| Leaky integrate-and-fire | $\begin{aligned} \frac{{dv}_{m}}{dt}=\frac{gL\left( EL-v_{m} \right)+g_{e}V_{unit} -g_{i}V_{unit} +I_{ext}(t,i)}{C}\# \end{aligned}$ |
| Synapse model | $\begin{aligned} \frac{dg}{dt}= \frac{-g}{tau}, tau=5 ms\# \end{aligned}$ |
| Adaptive exponential integrate-and-fire | $\frac{{dv}_{m}}{dt}=\frac{- gL\left( v_{m}- EL \right)+ \Delta_{T}gLe^{\frac{v_{m}-VT}{\Delta_{T}}}- w + g_{e}V_{unit} -g_{i}V_{unit} +I_{ext}(t,i)}{C}$ |
| Adaptation model | $\frac{dw}{dt}= \frac{a\left( v_{m}- EL \right) - w}{\tau_{w}}$ |

| Data analysis | |
| --- | --- |
| Name | Equation |
| Coherence | $\begin{aligned} C_{xy}=\frac{{\vert P_{xy}\vert}^{2}}{P_{xx}P_{yy}}\# \end{aligned}$ |
| Granger Causality | $\begin{aligned} Y_{t}=\sum_{i=1}^{n_{1}} \alpha_{i} Y_{t-i}+v_{t}\# \end{aligned}$  $\begin{aligned} Y_{t}=\sum_{i=1}^{n_{1}} \alpha_{i} Y_{t-i}+\sum_{i=p}^{n_{2}} \beta_{i} X_{t-i}+u_{t} \# \end{aligned}$  $\begin{aligned} F=log \left\{ \frac{var\left( v \right)}{var\left( u \right)} \right\}, information (bits)= {log}_{2}\left( F \right)\# \end{aligned}$ |
| Transfer Entropy | $\begin{aligned} T_{X\overset{\to}{}Y}=MI\left( Y_{t+1};X_{t}^{\left( k \right)} \vert Y_{t}^{\left( l \right)} \right) \end{aligned}$  $\begin{aligned} =\sum_{y_{t+1}, y_{t}^{\left( k \right)}, x_{t}^{\left( l \right)}} p(y_{t+1}, y_{t}^{\left( k \right)},x_{t}^{\left( l \right)}) log\frac{p\left( y_{t+1} \vert y_{t}^{\left( k \right)},x_{t}^{\left( l \right)} \right)}{p\left( y_{t+1} \vert y_{t}^{\left( k \right)} \right)} \# \end{aligned}$ |
| Mean Reconstruction Error | $\begin{aligned} RE=\frac{\sum Var\left( x(t)-\hat{x}(t) \right)}{\sum Var\left( x(t) \right)} \# \end{aligned}$ |
